# Impact Mechanism of Spectral Differentiation on PV Performance and Optimization of PV Systems in Shaded Forest Environments

**DOI:** 10.3390/s25237373

**Published:** 2025-12-04

**Authors:** Dongxiao Yang, Yuan He, Latai Ga, Daochun Xu, Xiaopeng Bai, Wenbin Li

**Affiliations:** 1 School of Technology, Beijing Forestry University, Beijing 100083, China; yangdx666@bjfu.edu.cn (D.Y.); heyuan96@bjfu.edu.cn (Y.H.); xudaochun@bjfu.edu.cn (D.X.); xiaopengbai@bjfu.edu.cn (X.B.); 2 State Key Laboratory of Efficient Production of Forest Resources, Beijing 100083, China; 3 Key Laboratory of National Forestry and Grassland Administration on Forestry Equipment and Automation, Beijing 100083, China; 4 School of Renewable Energy, Inner Mongolia University of Technology, Ordos 017010, China; galtai@imut.edu.cn

**Keywords:** understory spectral differentiation, shaded forest environment, silicon-based PV cells, PV system, forest monitoring sensors

## Abstract

**Highlights:**

**What are the main findings?**

- Shading spectra of distinct forest types exhibit significant differences in the 380–680 nm band, with a consistent variation trend above 680 nm.
- Poly-Si PV cells achieve optimal output performance under the shading spectral environments of different forest types in summer, and the designed circuit meets the stable operation requirements.

**What is the implication of the main finding?**

- This study fills the gap in shading spectra of northern summer arbor forests, explores their impacts on PV output characteristics, and provides a basis for selecting Poly-Si as forest PV materials.
- A scheme integrating PV material selection and energy harvesting circuits is proposed, offering a novel approach to addressing the power supply challenge of forest monitoring sensors.

**Abstract:**

The global low-carbon transition is driving the use of renewable energy for ecological monitoring. Traditional power supply for forest monitoring sensor equipment is constrained by high wired costs, frequent battery replacement, and the limitations of low light levels and special spectra under forest canopies on photovoltaic (PV) compatibility. Existing research lacks exploration of the correlation between under-forest spectra and PV performance. This study measured the summer understory light spectra of five tree species in Beijing, evaluated the performance of three types of PV cells—monocrystalline silicon, polycrystalline silicon, and amorphous silicon—and designed a low-light energy harvesting circuit. Results indicate that spectral differences under tree canopies are concentrated from 380–680 nm, exhibiting a distinctive forest-specific spectral feature of “high-band enrichment” above 680 nm. Under low-light conditions, polycrystalline silicon photovoltaics demonstrates optimal performance when adapted to this high-band spectrum. The designed circuit can activate at 5 W/m^2^ irradiance and stably output 4.16 V voltage. This study fills a spectral gap in northern summer tree canopies, providing a comprehensive solution of “material adaptation + circuit customization” for the practical deployment of shaded forest PV systems.

## 1. Introduction

With the increasing global emphasis on environmental protection and sustainable development, the development and utilization of renewable energy have become a key research area. PV technology, as an efficient and clean energy solution, has been widely adopted in various applications, including rooftop power generation, desert power plants, and traffic signal systems [1]. In forest environments, wireless sensor networks play a vital role in forest resource monitoring, ecological conservation, and early fire detection. However, traditional power supply methods face significant challenges. Wired power supply has the characteristics of difficult construction, high cost, and susceptibility to environmental damage, while battery-powered systems have limited capacity and difficult replacement, which restricts the long-term operation of sensor networks. Frequent battery replacement will increase maintenance costs and labor input and also cause environmental pollution. Additionally, battery life is affected by environmental factors such as temperature and humidity, further compromising the reliability and stability of sensor networks [2,3]. In recent years, the application of PV technology in forest environments has garnered significant attention. PV cells convert solar energy into electricity, providing clean power for wireless sensors. This reduces reliance on traditional batteries, lowers maintenance costs, and aligns with forest ecological conservation requirements [4,5]. However, the unique characteristics of the forest environment impose higher demands on the performance of PV cells.

Compared to open areas, shaded environments under forest canopies exhibit unique characteristics: firstly, lower light levels, as canopy cover significantly reduces total irradiance reaching the forest floor; secondly, poor light stability, with frequent fluctuations in light intensity due to changes in solar azimuth and leaf movement; thirdly, the spectral characteristics are complex. Due to differences in leaf structure, pigment content, and canopy density among various tree species, the spectral composition of scattered light in shaded areas varies considerably [6,7,8]. Furthermore, PV cells made from different materials inherently exhibit distinct spectral response characteristics [9,10], and fluctuations in light irradiance further amplify these differences. Accurately quantifying the spectral characteristics of the forest canopy and matching suitable PV materials are prerequisites for ensuring the long-term stable operation of micro-power consumption devices under forest cover. Existing research has primarily focused on optimizing device performance under open or standard light sources, with a lack of systematic experimental data on the coupling mechanism between shaded environment spectra and PV output, especially a quantitative comparison of shading spectra from different tree species [11,12,13,14,15,16]. Meanwhile, the reverse bias and hot spot effects caused by partial shading have been proven to threaten module reliability [17]. However, relevant studies have primarily focused on non-uniform illumination scenarios. For complete shading conditions without direct light spots and overall low light, the spectral-performance mapping rules and low-light energy collection strategies still need to be further explored [18,19,20].

During summer, lush foliage provides stable shade conditions and a rich spectrum of light, making it an ideal window for studying the performance of forest-based photovoltaics. In contrast, during spring and autumn, leaves grow and fall off frequently, leading to significant fluctuations in lighting conditions, making it difficult to ensure experimental repeatability and stability. In winter, due to the complete fall of leaves, lighting conditions become monotonous, preventing the acquisition of diverse spectral data, and thus, it is not suitable for conducting systematic research [21,22]. Therefore, summer is a crucial period for exploring the impact of different forest types and lighting environments on PV power generation performance. The research results hold significant scientific importance for optimizing forest-based PV systems and developing new adaptive materials.

Based on this, this article aims to achieve the following three specific goals: (1) to measure the shading spectral characteristics of five typical tree forests in Beijing under summer canopy; (2) systematically evaluate the output performance of three different materials of PV cells in real forest spectral environments; (3) design and validate an energy harvesting circuit suitable for low-light forest environments to support stable power supply for forest monitoring sensors. The research aims to fill the gap in spectral data of summer canopy shading in northern China, establish a technical path for collaborative optimization of a “spectrum material circuit”, and provide theoretical basis and engineering reference for the practical deployment of PV systems in forest areas.

## 2. Materials and Methods

### 2.1. PV Panels

PV cells are the core components of PV power generation systems, and their working principle is based on the PV effect: when sunlight strikes the surface of PV materials, the semiconductor absorbs the energy of photons. If the photon energy exceeds the material’s bandgap width, electrons in the valence band are excited and transition to the conduction band, forming electron–hole pairs. Under the influence of the built-in electric field within the PN junction, electrons and holes undergo directional separation, with electrons migrating towards the negative electrode and holes migrating towards the positive electrode, forming a stable potential difference. Upon connecting an external load, the photogenerated current continues to be output, completing the conversion of light energy into electrical energy [23].

Currently, the common PV cells on the market can be divided into three types based on the morphology of silicon materials: monocrystalline, polycrystalline, and amorphous. All of them use silicon as the basic material and possess high PV conversion efficiency and long service life. However, significant differences exist among the three in terms of crystal structure and optoelectronic properties, which directly determine their spectral response characteristics and applicable scenarios. Monocrystalline silicon photovoltaic (Mono-Si PV) cells are manufactured from single-crystal silicon ingots free of grain boundaries. Their highly ordered crystal structure enables high carrier mobility and lifetime, achieving PV conversion efficiencies exceeding 20%. However, their production process is complex and costly, and they exhibit sensitivity to variations in light intensity and ambient temperature [24]. The cell exhibits the highest quantum efficiency in the long-wave region (approximately 600–900 nm), with a spectral response range spanning from 400 to 1100 nm. However, its absorption coefficient is relatively weaker in the short-wave region (400–500 nm), and its spectral characteristics are also influenced by the optical properties of the encapsulation materials [23]. The quantum efficiency of polycrystalline silicon photovoltaic (Poly-Si PV) cells slightly decreases in the short-wave region, but they still maintain a PV conversion capability similar to that of Mono-Si in the mid-to-long-wave region (approximately 700–900 nm), with a more gradual decrease on the long-wave side [25]. Amorphous silicon photovoltaic (a-Si PV) cells are fabricated using thin-film technology, where a-Si thin films are deposited on a substrate. They exhibit a high absorption coefficient for short-wavelength light (approximately 400–600 nm) but suffer from low carrier mobility and initial conversion efficiency. However, these cells offer advantages such as low silicon material consumption, low energy consumption during fabrication, light weight, and good flexibility, making them suitable for applications where weight and flexibility are highly critical [26]. a-Si PV cells have a wider bandgap, with their quantum efficiency peak located in the green region (around 500 nm). When the wavelength exceeds 580 nm, the absorption coefficient drops rapidly, and the response above 800 nm can be neglected. The effective spectral window is significantly narrower than that of crystalline silicon cells [27]. Mono-Si PV cells exhibit the widest spectral response and the highest conversion efficiency, followed by Poly-Si PV cells, which offer an outstanding cost-performance ratio. a-Si PV cells, on the other hand, are concentrated in the visible blue–green region and excel in terms of their thinness, lightness, and flexibility. In environments rich in long-wavelength light, crystalline silicon PV cells demonstrate significant advantages, whereas a-Si PV cells rely on short-wavelength photons for conversion. In practical applications, factors such as spectral distribution, cost, and installation conditions must be comprehensively considered to select the most suitable type of PV cell.

The aforementioned studies on PV cell performance have mostly been conducted in open environments or under ideal laboratory conditions, lacking systematic research on the actual environmental conditions of forest shading with its time- and space-varying spectral characteristics. After being absorbed and scattered by the canopy, the spectral composition of understory light differs significantly from that of open land. This variation will inevitably have distinct impacts on the photogenerated carrier processes of different PV cells. However, there is still a lack of systematic experimental data on the coupling mechanism of “differentiated understory spectrum–PV output”. To reveal the quantitative relationship between different forest lighting environments and the output characteristics of various PV cells, and to provide a basis for the selection and optimization of PV modules within forests, this paper selects Mono-Si PV, Poly-Si PV, and a-Si PV cells, which are commonly found on the market, as the research objects for experiments. During the experiment, the PV cells selected for use had consistent dimensions of 50 × 50 × 3 mm, in order to eliminate interference from size differences on output power and ensure that the experimental variables were solely related to environmental lighting characteristics and the cell materials themselves, thereby enhancing the reliability of the results. Detailed parameters of the three types of PV cells are shown in Table 1.

### 2.2. Mathematical Model of PV Cells

**Figure 1:**
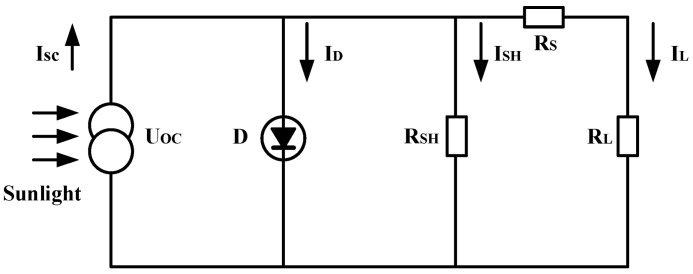
Equivalent circuit diagram of PV cells.

Currently, there are numerous mathematical models for equivalent circuits of PV cells, among which the single diode model is the simplest and most commonly used equivalent model for PV cells, which can effectively describe the response characteristics of PV cells. Considering that all experiments in this study were conducted in a completely shaded environment with overall low light and no local bright spots, the irradiance difference between PV cell panels is minimal, and there is no risk of reverse bias and hot spots caused by local shading. Therefore, a single diode is used to model the PV cell, and its equivalent circuit is shown in Figure 1.

From the above figure, we can conclude:

(1) $$I_{L}=I_{SC}-I_{D}-I_{SH}$$

In the formula, $I_{SC}$ represents the output current when the external resistance is 0 or the external circuit is short-circuited under standard test conditions, which is related to PV cell temperature, area, and light intensity; $I_{D}$ represents the total diffusion current generated by the diode due to light on the PN junction; the specific formulas for $I_{SC}$ and $I_{D}$ are as follows:

(2) $$I_{D}=I_{0}(e^{\frac{qE}{AKT}}-1)$$

(3) $$I_{SH}=\frac{U_{L}+I_{L0}R_{S}}{R_{SH}}$$

Therefore, the output current $I_{L}$ of the PV cell can be expressed as follows:

(4) $$I_{L}=I_{SC}-I_{0}(e^{\frac{qE}{AKT}}-1)-\frac{U_{L}+I_{L0}R_{S}}{R_{SH}}$$

Under ideal conditions, the series resistance $R_{S}$ of the PV cell is very small, while the shunt resistance $R_{SH}$ is very large. Both can be neglected in calculations, and thus the output current I_L_ of the cell can be further simplified as follows:

(5) $$I_{L}=I_{SC}-I_{0}(e^{\frac{qE}{AKT}}-1)$$

From this, the expression for the output power of the PV cell can be derived as follows:

(6) $$P={U_{L}I_{L}=U_{L}I}_{SC}-{U_{L}I}_{0}(e^{\frac{qE}{AKT}}-1)$$

(7) $$U_{L}=\frac{AKT}{q}\ln\left(\frac{I_{SC}+I_{L}}{I_{0}}+1\right)$$

When the external resistance is infinitely large or the external circuit is open, the output voltage $U_{OC}$ is related to the light intensity but not to the area of the PV cell. Its expression is as follows:

(8) $$U_{L}=\frac{AKT}{q}\ln\left(\frac{I_{SC}}{I_{0}}+1\right)\approx \frac{AKT}{q}\ln\left(\frac{I_{SC}}{I_{0}}\right)$$

The output characteristics of PV cells are non-linear. When the output power reaches its maximum, the corresponding voltage and current are the maximum power point voltage and maximum power point current, respectively. The power generation of PV cells increases with the increase in cell voltage. When the output power grows to the rated output power, it reaches a critical point. After that, if the cell voltage continues to increase, the cell current and power will rapidly decrease to 0.

The meanings of the electrical parameters in the aforementioned PV cell mathematical model are presented in Table 2.

### 2.3. Power Management Module

To build an energy harvesting system capable of long-term stable operation in low-light forest environments, a comprehensive power management solution tailored to the system’s requirements is still necessary in addition to PV cells. Due to interference from the complex forest environment, PV cells exhibit low output voltage and significant fluctuations. Traditional PV systems struggle to start up under low voltage input, making it impossible to provide stable power supply for forest monitoring equipment. In addition, traditional solutions lack the ability to dynamically adjust load, making it difficult to maximize the energy conversion efficiency of PV cells [28,29,30]. This study designed an ultra-low-voltage-start PV energy harvesting circuit featuring maximum power point tracking, buck–boost conversion, voltage regulation, and overvoltage/undervoltage protection.

**Figure 2:**
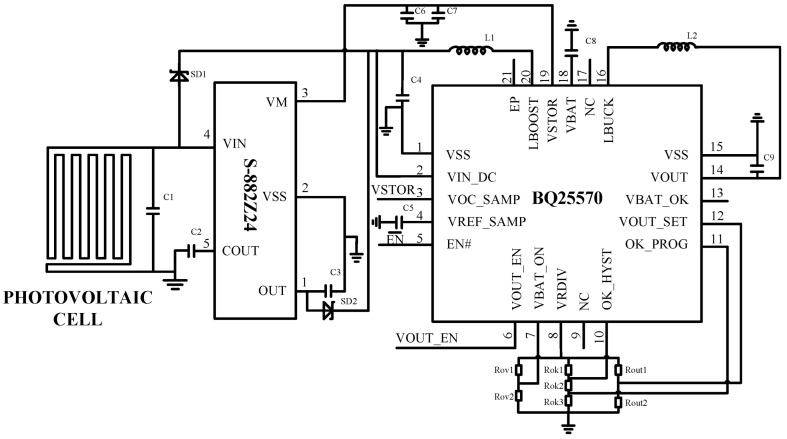
Design diagram of power management module.

**Figure 3:**
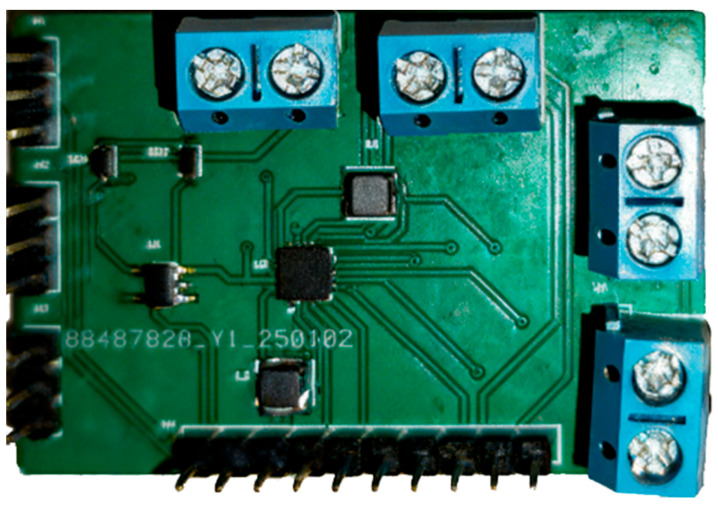
Schematic diagram of the energy harvesting circuit.

Figure 2 shows the schematic diagram of the circuit module design. Specifically, this circuit centers on the S-882Z24 chip and employs a boost circuit that can initiate operation when the PV cell output voltage drops as low as 300 mV, while maintaining operation at 100 mV through a negative feedback control mechanism. Using the BQ25570 chip with MPPT function, the built-in algorithm dynamically adjusts the load impedance to ensure that the PV cell operates at its maximum power point. A synchronous rectification DC-DC converter is employed to stabilize the input voltage from 0.1 to 5 V to an output voltage of 4.2 V, satisfying the power supply requirements of forest monitoring equipment under specific conditions. This design adopts a low-power chip energy management strategy, which can minimize energy loss through feedback control. It also incorporates overvoltage and undervoltage protection functions to safeguard the energy storage battery and ensure system stability. The physical circuit is shown in Figure 3.

### 2.4. Experimental Setup and Uncertainty Analysis

**Figure 4:**
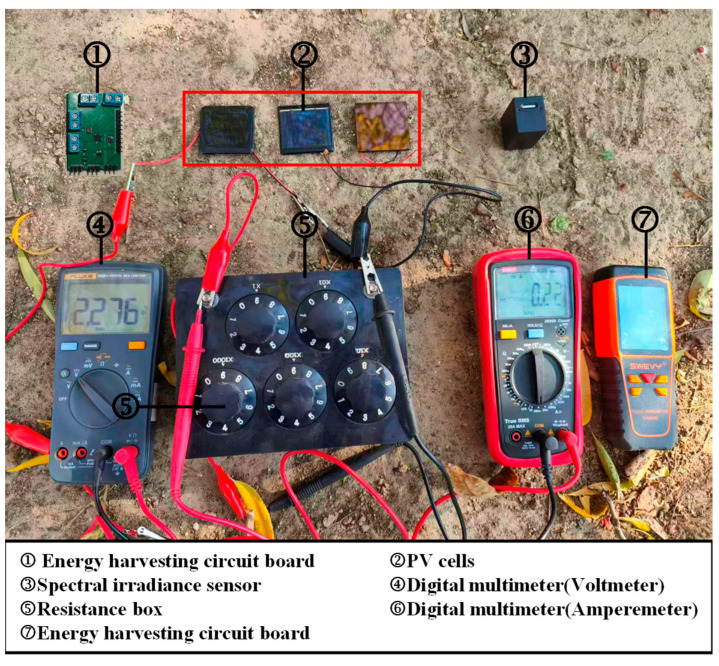
Experimental setup. From left to right in ②, the devices are the Mono-Si PV cell, Poly-Si PV cell, and a-Si PV cell, respectively.

The data collected during the experiment primarily consists of irradiance intensity in shaded environments, load resistance values, as well as the output voltage and current of PV cells. The experimental setup is shown in Figure 4, and the detailed technical specifications of the measuring instruments are listed in Table 3. The test environment temperature was maintained at 27 ± 1 °C. Both the PV cell and the spectral irradiance sensor were placed perpendicular to the ground, with their surfaces parallel. Data was sampled at an interval of 5 s, and the average value was calculated from three consecutive recordings.

To ensure the accuracy of experimental results, an uncertainty analysis of the measured parameters is required. The error of directly measured parameters depends on the accuracy of the measuring instruments. In this experiment, the directly measured parameters mainly include environmental irradiation intensity, voltage, and current. As an indirect parameter, the measurement error of power is influenced by the measurement errors of voltage and current. Using the method proposed by Akpinar [31], the overall experimental uncertainties for irradiation intensity, voltage, current, and power, as calculated, are 4%, 0.53%, 0.53%, and 0.75%, respectively. All these uncertainties are less than 5%, which is within an acceptable range.

## 3. Results and Discussion

### 3.1. Analysis of Light Environments in Different Forest Types

To investigate the output performance of PV cells under different forest shading conditions during summer, this study first measured irradiance data under various environmental conditions in five common tree forests in northern China. The measurement work was conducted from 12:00 to 14:00 during the noon period from 15 May to 30 May 2025, covering different areas of Beijing Olympic Forest Park (40°01′ N, 116°23′ E) and Beijing Forestry University (39°55′ N, 116°25′ E). The study selected forest areas of *Populus canadensis* (*P. can*), *Paulownia occidentalis* (*P. occ*), *Juniperus rigida* (*J. rig*), *Pinus tabulaeformis* (*P. tab*), and *Eucommia ulmoides* (*E. ulm*), randomly selecting five areas within each forest type and measuring irradiance under three conditions: clear sky transmitted light area, clear sky forest shadow area, and cloudy day shadow area. The results showed that the irradiance in the transmitted light area was higher than that in the shaded area across all forest types. However, there were significant fluctuations in the irradiance of the transmitted light area, with a limited and unstable distribution. These areas are primarily concentrated in the gaps between trees, which are susceptible to the changing trajectory of the sun and the swaying of tree branches and leaves. In comparison, the irradiance levels in shaded areas on sunny and cloudy days are relatively close. Despite the lower irradiance in shaded areas, its fluctuation is smaller, distribution is broader, and stability is stronger, making it the primary lighting environment in forest settings. Detailed measurement data is provided in Table 4.

In the forest environment, although transmitted light has a relatively high intensity, its instability and limited range make it difficult to serve as the primary source of light energy. Therefore, the following content is based on the experimental exploration conducted in the shaded forest environment on sunny days.

### 3.2. Study on Spectral PV Response Mechanisms of Different Forest Types

**Figure 5:**
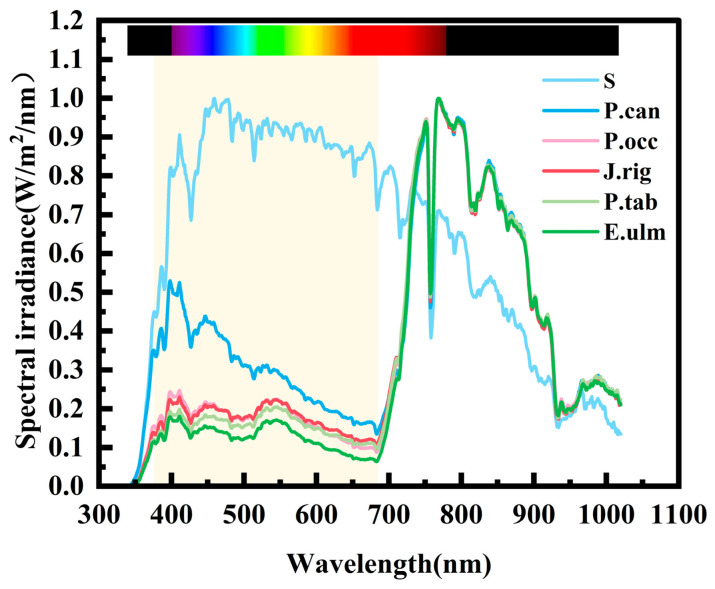
Comparison of spectra in different forest shade environments.

To explore the spectral characteristics of shadows in different forest interiors during summer and their impact on the power generation performance of different PV cells, this study measured the spectra of scattered light in the shadows of the aforementioned five types of arbor forests on a sunny day, as well as the spectra in an open area on the same sunny day (S). After normalization, the results were compared, and the specific results are shown in Figure 5 below. The measurement was conducted during the noon period from 12:00 to 14:00 on a typical sunny day in summer, when the lighting conditions are most representative. During the measurement, a high-precision spectral sensor was used, with an average irradiance intensity of 7.23 W/m^2^ and a control error within 0.5 W/m^2^. The irradiance intensity was kept as consistent as possible to minimize potential interference from variations in light intensity on the spectral measurement results.

As shown in Figure 5, the spectral characteristics of shaded areas in different forest types during summer exhibit significant differences from those in open areas. The open-area spectrum forms a distinct peak between 400 and 680 nm (violet to orange-red, the core range of visible light), with intensity gradually decreasing beyond 700 nm as wavelength increases. This pattern is associated with atmospheric scattering attenuation and absorption by water vapor and carbon dioxide, a relationship confirmed by atmospheric optical studies [32,33,34,35]. In contrast, the spectra of the five forest types exhibit distinct segmented characteristics: the intensity from 400 to 680 nm is significantly lower than that in open areas, with low-intensity distribution in blue, green, and orange-red light, reaching its lowest point around 680 nm, and then rapidly rising to around 770 nm (the starting point of near-infrared) to form a peak; although it gradually decays from 770 to 940 nm, the relative intensity is still higher than that in open areas; after 940 nm, it continues to decay, gradually converging with the infrared spectrum of open areas. The formation of this characteristic may be closely related to the optical properties of vegetation and the mechanism of light propagation. Photosynthetic pigments such as chlorophyll and carotenoids in vegetation leaves have strong absorption characteristics for light in the 400~680 nm wavelength band [36]. Through photosynthesis, they convert light energy into chemical energy, resulting in a significant decrease in light intensity in this wavelength band within the forest. Upon entering the wavelength band of 680~770 nm, the reflectance of vegetation leaf cell structures gradually increases, while absorption significantly decreases. Consequently, the light intensity rapidly climbs from the lowest point at 680 nm to a peak around 770 nm. After 770 nm, the reflection effect of vegetation gradually weakens, and the light intensity gradually decays. However, in the range of 770~940 nm, the residual effect of vegetation reflection is still higher than that in open areas [37,38,39]. After 940 nm, the strong absorption effect of water vapor in the atmosphere becomes dominant, and the attenuation trend of forest spectra tends to be consistent with that in open areas, which is consistent with the absorption law of the atmosphere towards infrared light [34,35]. The spectral differences among shaded areas of various tree species are primarily concentrated within the wavelength range of 380~680 nm. Based on the proportion of this wavelength band, the order from highest to lowest is *P. can > P. occ > J. rig > P. tab > E. ulm*. This may be associated with variations in the pigment content, thickness, and canopy structure of the tree species’ leaves. These spectral characteristics offer clear environmental variables for analyzing the performance response of PV cells, laying a foundation for subsequent experiments.

Due to the inherent differences in the absorption efficiency of PV cells with different materials for light in specific wavelength bands, the aforementioned spectral differentiation will inevitably lead to regular differences in their power generation performance. Therefore, based on the aforementioned experimental conditions, the experimental data recorded is presented in the table below.

Combining the spectral characteristics of different shaded forests and the maximum output power data of the three types of cells in Table 5, the PV performance in the five forest types exhibits a clear pattern: Poly-Si PV cells have the overall best output power, which is higher than that of Mono-Si PV cells except in the *J. rig* forest; the output power of both Mono-Si and Poly-Si PV cells is significantly higher than that of a-Si PV cells in all forest types, and the power output of the a-Si PV cell is always the lowest, with the gap between it and the former two remaining stable as the environment changes. From the perspective of the correlation between spectrum and power, the spectral differences of the five forest types are concentrated in the 380–680 nm low-wavelength band, showing a gradual decrease in the proportion of this band from the *P. can* forest to the *E. ulm* forest. This feature strictly corresponds to the change in cell power, that is, the power of Mono-Si PV cells and Poly-Si PV cells gradually increases as this low waveband decreases (the slight deviation of Mono-Si PV cells in the *P. tab* forest does not change the overall trend), while that of a-Si PV cells continues to decrease as it decreases.

The formation of the aforementioned patterns stems from the synergistic interaction between the inherent properties of the material and the unique spectral environment within the forest. From a material perspective, crystalline silicon materials (Mono-Si, Poly-Si) have demonstrated high-efficiency response characteristics to light in the high-wavelength band above 680 nm, whereas the spectral response of a-Si is concentrated in the low-wavelength band between 380 and 680 nm [9,27]. From an environmental perspective, this study’s empirical findings reveal that, in summer, higher-wavelength light above 680 nm is more concentrated in shaded environments compared to open areas. Furthermore, as one moves from *P. can* to *E. ulm* forests, the proportion of low-wavelength light (380~680 nm) decreases, while the relative proportion of higher-wavelength light increases. This environmental spectral characteristic is precisely compatible with the response properties of crystalline silicon materials: when the proportion of the low-wavelength band decreases, the utilization of light in the core response band (>680 nm) by Mono-Si and Poly-Si remains undisturbed, and their output power even improves. In contrast, since a-Si relies on light in the low-wavelength band, its output power decreases as the proportion of this band declines. This mechanism is fully consistent with the inherent spectral response of different materials and the measured spectral characteristics of forest shade, providing a direct experimental basis for the optimized selection of PV systems in forest environments.

### 3.3. Forest Canopy Low-Irradiance PV Response Characteristics

In the shaded forest environment, the power generation performance of PV cells is influenced not only by spectral characteristics but also by light irradiance intensity [24,40]. Variations in light irradiance directly affect the output power of PV cells, thereby impacting the efficiency and stability of the entire PV system [41,42]. Therefore, to comprehensively assess the power generation performance of different types of crystalline silicon PV cells in shaded forest environments, this study further investigates the impact of light irradiance on the power generation of three types of crystalline silicon PV cells.

**Figure 6:**
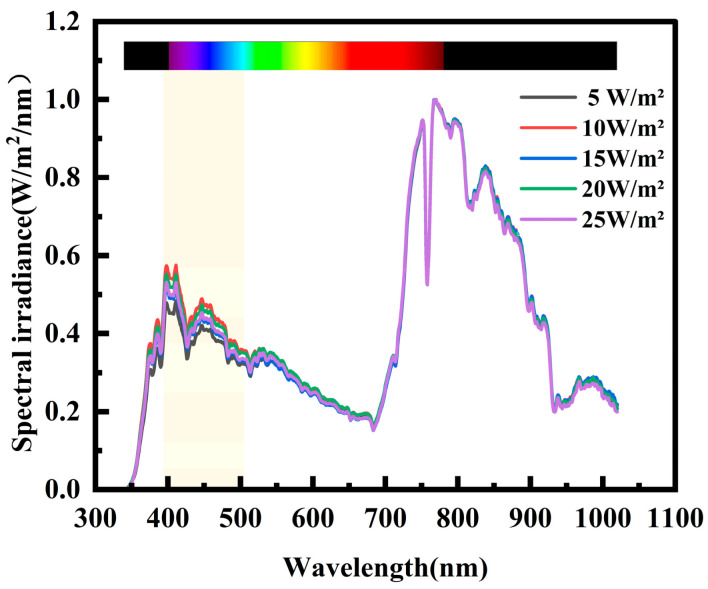
Spectra at different irradiance levels in a *J. rig* forest.

**Figure 7:**
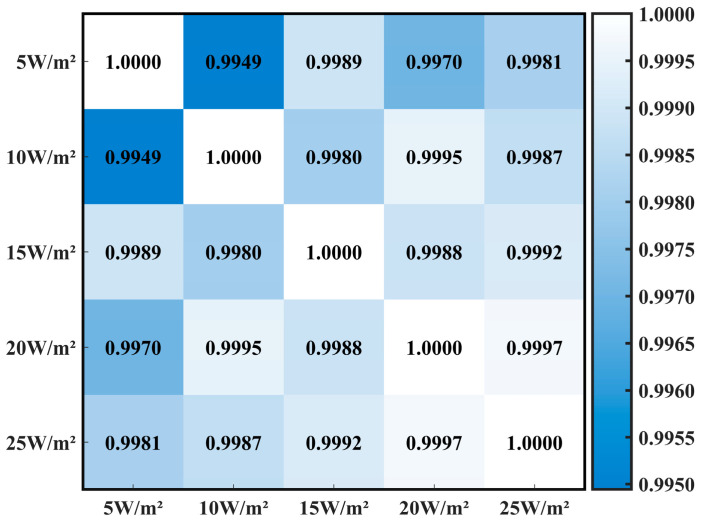
Pearson correlation coefficient heatmap.

To accurately assess the impact of varying light irradiance levels on the power generation of three types of crystalline silicon PV cells, it is crucial to maintain consistent spectral characteristics of the shaded forest environment throughout the experiment, with light irradiance intensity as the sole variable. To achieve this, the study selected five common arbor forests in northern China and measured spectral characteristics data under five distinct irradiance levels in each forest. The spectral data were normalized. For instance, the normalized spectral characteristics curve for the *J. rig* forest is depicted in Figure 6. Within the same forest, the spectral characteristic curves under different irradiance levels are essentially identical across most wavelengths, with only the low-wavelength band (400–500 nm) showing slight fluctuations. To ensure the rigor of the experimental results, Pearson correlation coefficients were calculated between spectral data under different irradiance levels within the same forest. Taking the *J. rig* forest as an example, the Pearson correlation coefficient heatmap is shown in Figure 7. It can be seen that the Pearson correlation coefficients between spectra of the same tree species under different irradiance levels are as high as 0.99 or above, indicating a high degree of correlation. Therefore, it can be concluded that changes in irradiance under the shade of the same tree species do not affect changes in its spectral characteristics.

**Figure 8:**
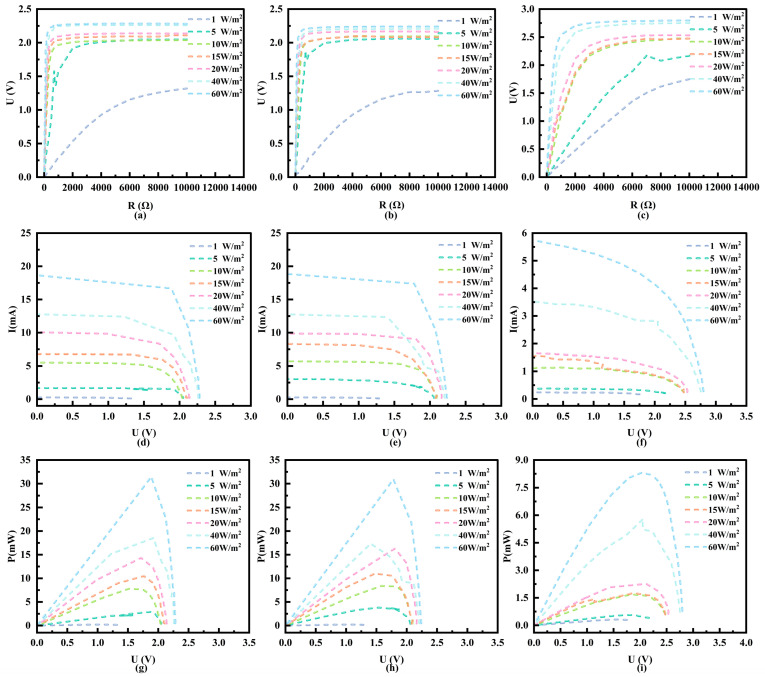
Output characteristic curves of three types of PV cells. (**a**–**c**) show the irradiance–output voltage–load characteristic curves of Mono-Si, Poly-Si, and a-Si PV cells, respectively; (**d**–**f**) represent the irradiance–output current–output voltage (I-V) characteristic curves of the aforementioned Mono-Si PV, Poly-Si PV, and a-Si PV cells; (**g**–**i**) denote the irradiance–output power–output voltage (P-V) characteristic curves of the three PV cells.

Next, power generation experiments were conducted using the aforementioned three different types of PV cells. The experiment was carried out in a *J. rig* forest with high tree density in the Beijing area of northern China (39°55′ N, 116°25′ E), which features a typical shaded and diffused light environment. The experiment was conducted at noon on 29 May 2025. To ensure the reliability of the experimental results, while ensuring consistency in test spectra, we measured the output characteristics of PV cells made of three different materials under varying light irradiance in a shaded environment using a multimeter and a variable resistor box. The output characteristic curves are plotted as shown in Figure 8. Due to the constraints of the forest experimental setting, it was challenging to maintain a constant change in irradiance. Therefore, seven sets of typical data were selected based on actual environmental conditions, with irradiances of 1 W/m^2^, 5 W/m^2^, 10 W/m^2^, 15 W/m^2^, 20 W/m^2^, 40 W/m^2^, and 60 W/m^2^, respectively. The error fluctuation was controlled within ±1 W/m^2^.

The experimental results indicate that, in a shaded environment, the output voltage of Mono-Si PV, Poly-Si PV, and a-Si PV cells increases as the load resistance increases. Once the load resistance reaches a certain threshold, the output voltage stabilizes and saturates. Prior to this saturation point, for a given load resistance, the output voltage increases with the increase in irradiance. A comparative analysis of three types of PV cells reveals that, under low light irradiance conditions, Mono-Si and Poly-Si exhibit lower saturation internal resistance compared to a-Si cells. The output voltage of both Mono-Si PV and Poly-Si PV cells can reach its maximum under relatively low external load resistance, with minimal difference between the two. In contrast, a-Si PV cells have higher internal resistance but can output relatively higher voltage.

From the irradiance–I-V characteristic curves (Figure 8d–f) and the irradiance–P-V characteristic curves (Figure 8g–i), it can be seen that, under forest shading conditions, all three types of PV cells are capable of performing PV conversion in shaded environments without direct sunlight, possessing a certain power generation capability. Their output current and maximum power increase with the increase in environmental irradiance. When the environmental irradiance is constant and the output voltage has not yet reached saturation, the output current of the PV cell remains essentially unchanged as the output voltage varies, exhibiting I-V characteristics similar to that of a current source. The aforementioned experimental results quantify the variation of dynamic impedance with irradiance under shaded conditions, laying a data foundation for the development of dedicated MPPT control strategies for low-light environments in forests.

**Figure 9:**
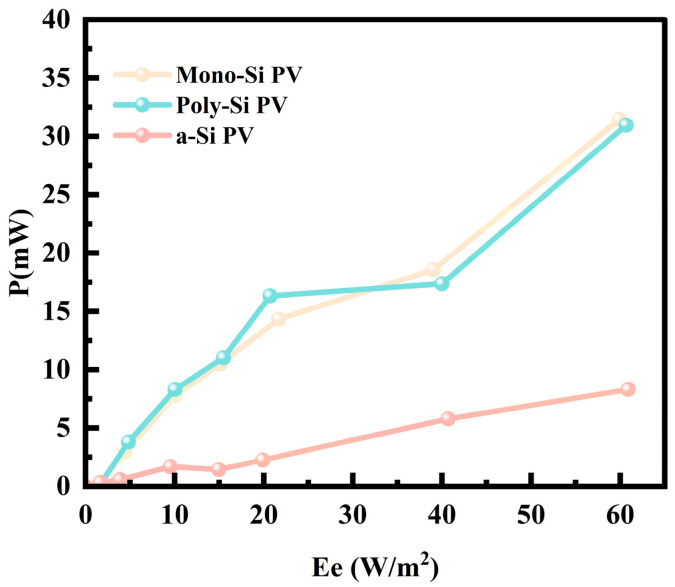
Irradiance–maximum output power characteristic curves.

To visually compare the relationship between the maximum output power of three types of PV cells and environmental irradiance under different irradiance levels in shaded and low-light environments, the maximum output power of each type of cell under different irradiance levels was extracted, and an irradiance–maximum output power characteristic curve was plotted, as shown in Figure 9. It can be seen that, in the same environment, the maximum output power of the three types of PV cells increases with the increase in environmental irradiance. Compared with a-Si PV cells, Mono-Si PV and Poly-Si PV cells can output higher power at lower irradiance, and as the irradiance increases, their output power increases at a faster rate. Upon further comparison between Mono-Si PV and Poly-Si PV cells, it was observed that, when the environmental irradiance is below 35 W/m^2^, the maximum output power of Poly-Si PV cells is slightly higher than that of Mono-Si PV cells. However, as the environmental irradiance continues to increase, the maximum output power of Poly-Si PV cells gradually becomes lower than that of Mono-Si PV cells. This phenomenon may be attributed to the fact that the crystal structure of Poly-Si is composed of numerous small grains, and grain boundaries and defects can enhance light scattering and absorption. Especially under low irradiance conditions, these characteristics are conducive to capturing more scattered light, thereby improving the power generation efficiency of photogenerated carriers and exhibiting better power generation capability under weak light conditions [25,43,44,45]. In comparison, the Mono-Si crystal structure is more perfect, with fewer defects and impurities, enabling more efficient utilization of photogenerated carriers under high irradiance conditions, thus exhibiting higher output power under high irradiance [36,46,47].

Based on the measured characteristic data of the inter-forest lighting environment in Table 4, it can be clearly divided into three scenarios: transmitted light, sunny day shadows, and cloudy day shadows. Although transmitted light has a high irradiance, some of which can reach 87–349 W/m^2^, it is only concentrated in tree gaps, has a limited distribution, and fluctuates violently, making it not the main light source in the forest. The irradiance in sunny and cloudy day shadow areas, which cover a wider range and have a more stable state within the forest, generally remains below 20 W/m^2^. In this section, the irradiance exceeding 30 W/m^2^ only appears at the forest edge, which is relatively rare and cannot represent the general lighting environment in the forest. It can be seen that the advantageous range of Poly-Si PV cells matches the shadowy lighting environment in the forest, with optimal performance, and its manufacturing cost is lower than that of Mono-Si. Therefore, Poly-Si PV cells are determined to be the best solution for powering forest monitoring equipment in shaded forest environments.

### 3.4. Verification Experiment of Micro-Energy Collection Circuit

In complex forest environments, the stability of PV cell output voltage and current is constrained by multiple factors. Although Poly-Si exhibits superior power generation performance under low-light conditions, its output power remains unstable due to factors such as light fluctuations and natural interference. Therefore, this study designed an ultra-low voltage start-up PV energy harvesting circuit. To verify the actual performance of the circuit in a shaded environment, the above-mentioned Poly-Si PV cells were used for power generation testing in the *J. rig* forest at noon on 3 June 2025, and their output characteristics were measured under different light irradiance levels in clear forest shade. During the testing process, different light irradiance levels in the shade of *J. rig* forest were selected as a single variable, and the range of environmental light irradiance variation was measured from 5 W/m^2^ to 25 W/m^2^, with a gradient of 5 W/m^2^ and an error not exceeding 0.5 W/m^2^.

**Figure 10:**
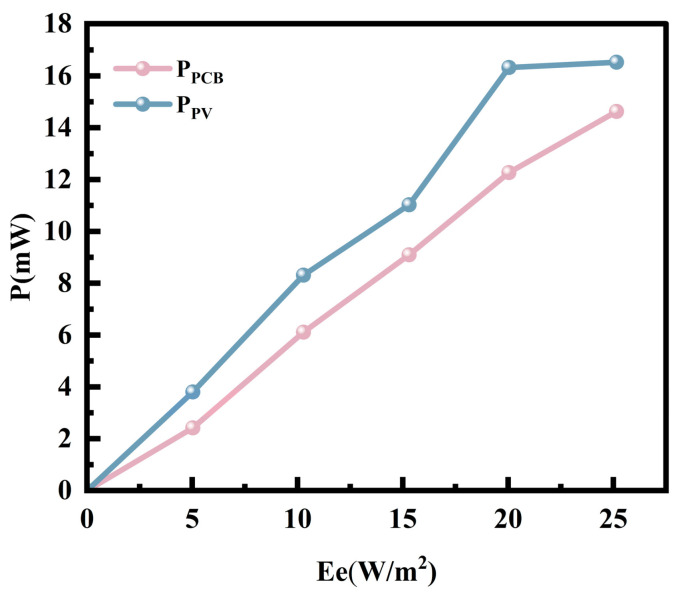
Comparison of maximum output power.

During the measurement process, the circuit can stably output a voltage greater than 4.16 V, and the output current increases with the increase in light irradiation intensity. Figure 10 shows a comparison of the maximum power output curves for Poly-Si PV modules with and without the PV energy harvesting circuit connected. It can be observed that the energy harvesting circuit effectively tracks the actual maximum power output even under low-light conditions as low as 5 W/m^2^. Compared to the unconnected state, it demonstrates a more stable power output curve across different irradiance levels. Based on previous research results, the micro-energy harvesting circuit, with its low-light adaptability and MPPT function of the selected chip, effectively solves the key problem of transitioning from “being able to generate electricity” to “being able to provide stable power supply”. The integrated “Poly-Si PV cells + ultra-low-voltage energy harvesting circuit” system meets the core requirements of PV power supply in shaded forest environments, providing a comprehensive solution of “material adaptation + circuit customization” for the actual deployment of shaded forest PV systems.

In summary, the understory lighting environment is not simply “weak light” but a special lighting condition with significant spectral selectivity. This study not only reveals the differential characteristics of shading spectra of different tree species but also elucidates the decisive role of the spectral response of PV materials and the matching degree of understory spectra in actual output performance. This discovery suggests that, in low-light applications such as ecological monitoring, PV materials should not be selected solely based on conversion efficiency under standard testing conditions but should be optimized based on on-site spectra. Future work can further combine seasonal dynamics with sensor power consumption models to achieve precise coordination between energy supply and load demand.

## 4. Conclusions

The global low-carbon transformation urgently requires cable-free ecological monitoring. However, the low light and differentiated spectra under forests make it difficult to adapt PV power supply, and forest sensors are still constrained by the high cost of wired and frequent battery maintenance. In this study, in situ experiments were conducted under five typical arbor forests in Beijing during summer. For the first time, it is revealed that the spectral differences among tree species are concentrated in the 380–680 nm band, with high-band enrichment above 680 nm, filling the gap in shaded spectra of northern arbor forests in summer. The irradiance in understory shaded environments is generally below 35 W m^−2^. Poly-Si PV cells have better output performance than a-Si and Mono-Si PV cells due to their high band gain matching and enhanced grain boundary scattering. The constructed low-light energy harvesting circuit can start at 5 W m^−2^ and stably output 4.16 V, solving the problem of low-light power supply under forests.

This study provides a comprehensive solution of “material adaptation + circuit customization” for shading forest PV systems by analyzing spectral characteristics, revealing PV response mechanisms, and designing power supply schemes. However, all experiments were conducted under complete shading conditions in five types of tree forests in Beijing during the summer, without involving local shading scenarios and lacking systematic data on multiseason, multiclimate zones and complex forest stands. In the future, it is necessary to establish a long-term observation network across seasons and climate zones, synchronously collect environmental data, and build a controllable local shading platform to systematically evaluate the impact mechanism of reverse bias and hot spot effects on the lifespan of forest-based PV systems in order to design targeted energy management strategies and promote the large-scale application of PV in ecological monitoring.

## Figures and Tables

**Table 1:** Parameters of three types of PV cells.

Parameter Name	Mono-Si PV	Poly-Si PV	a-Si PV
Open-circuit voltage (V_oc_, V)	2.4	2.4	2.8
Short-circuit current (I_sc_, mA)	176	143	42
Maximum power voltage (V_mpp_, V)	2	2	2.2
Maximum power current (I_mpp_, mA)	160	130	30
Maximum power (P_mpp_)	320	260	66
Fill factor (FF)	0.75–0.84	0.70–0.78	0.47–0.67
Conversion efficiency (η, %)	25	17.5	7
Temperature coefficient (TC, %/°C)	−0.40	−0.38	−0.25

Note: The Mono-Si and Poly-Si PV modules used in this study are commercial products composed of multiple series-connected cells. The Voc values listed in the table represent the total open-circuit voltage after series connection. For a-Si PV modules, the higher Voc is due to the intrinsic properties of thin-film structures.

**Table 2:** Electrical parameters of PV cell mathematical model.

Para	Meaning	Para	Meaning
$I_{L}$	PV cell output current	A	PN junction curve constant
$I_{SC}$	PV cell short-circuit current	K	Boltzmann constant (1.38 × 10^−23^ J/K)
$I_{D}$	Diode current	T	Temperature of PV cell
$I_{SH}$	PN junction leakage current	$U_{L}$	Output voltage
$I_{0}$	PN junction reverse saturation current	$R_{S}$	PV cell series resistance
q	Electron charge (1.6 × 10^−19^ C)	$R_{SH}$	PV cell shunt resistance
E	PN junction barrier height	D	Crystal diode

**Table 3:** Technical specifications of data measuring instruments.

Instrument Name	Model Number	Range and Accuracy
Spectral irradiance sensor	B42B5K10234NBPD	340–1020 nm, 0–3000 W/m^2^, ±4%
Temperature sensor	SW-6056	0–45 °C, ±1 °C
Digital multimeter (voltage)	Fluke 15B MAX01	0.1 mV–1000 V, ±(0.5% + 3)
Digital multimeter (current)	UT39E+	0.1 μA–20 A, ±(0.5% + 5)
Rotary resistance box	ZX21	0.1–99,999 Ω, ±1%

**Table 4:** Light irradiance of different tree species under various environmental conditions (Unit: W/m^2^).

Tree Species	Environmental Conditions	Area 1	Area 2	Area 3	Area 4	Area 5
	Sunny Transmitted Light	87.49	66.56	233.49	280.89	88.43
*P. can*	Sunny Shaded Light	13.64	16.56	16.40	17.54	17.74
	Overcast Shaded Light	9.43	11.51	14.35	13.61	11.92
	Sunny Transmitted Light	58.53	64.46	348.93	124.80	84.92
*P. occ*	Sunny Shaded Light	11.56	10.97	11.66	10.90	11.95
	Overcast Shaded Light	8.81	6.60	7.10	7.42	6.74
	Sunny Transmitted Light	135.00	268.07	109.04	167.45	149.14
*J. rig*	Sunny Shaded Light	12.16	14.24	12.94	12.37	12.20
	Overcast Shaded Light	6.43	6.36	6.63	5.50	5.59
	Sunny Transmitted Light	85.28	155.82	226.41	28.01	61.25
*P. tab*	Sunny Shaded Light	12.36	9.40	10.99	10.50	12.09
	Overcast Shaded Light	4.55	5.21	5.26	6.51	5.86
	Sunny Transmitted Light	45.83	102.11	48.23	339.64	204.58
*E. ulm*	Sunny Shaded Light	6.53	3.65	4.15	5.45	5.10
	Overcast Shaded Light	1.77	5.51	4.82	5.77	4.69

**Table 5:** Maximum power point output data of three types of crystalline silicon solar cells under different forest shade conditions.

Tree Species	Solar Cell Type	R (Ω)	U (V)	I (A)	P_max_ (mW)
	Mono-Si PV	400	1.532	3.860	5.914
*P. can*	Poly-Si PV	400	1.787	4.130	7.380
	a-Si PV	4000	1.705	0.420	0.716
	Mono-Si PV	300	1.564	4.050	6.334
*P. occ*	Poly-Si PV	300	1.701	4.400	7.484
	a-Si PV	5000	1.705	0.330	0.563
	Mono-Si PV	200	1.528	5.360	8.190
*J. rig*	Poly-Si PV	300	1.543	4.990	7.700
	a-Si PV	5000	1.523	0.330	0.503
	Mono-Si PV	300	1.683	4.350	7.321
*P. tab*	Poly-Si PV	300	1.654	5.170	8.551
	a-Si PV	5000	1.567	0.320	0.501
	Mono-Si PV	200	1.557	5.380	8.377
*E. ulm*	Poly-Si PV	200	1.566	6.090	9.537
	a-Si PV	7000	1.802	0.260	0.469

## Data Availability

Dataset available on request from the authors.
